# Changes in Sleep Time and Sleep Quality across the Ovulatory Cycle as a Function of Fertility and Partner Attractiveness

**DOI:** 10.1371/journal.pone.0092796

**Published:** 2014-04-07

**Authors:** Brooke N. Gentle, Elizabeth G. Pillsworth, Aaron T. Goetz

**Affiliations:** 1 Department of Psychology and Social Behavior, University of California Irvine, Irvine, California, United States of America; 2 Department of Anthropology, California State University, Fullerton, Fullerton, California, United States of America; 3 Department of Psychology, California State University, Fullerton, Fullerton, California, United States of America; University of Goettingen, Germany

## Abstract

Research suggests that near ovulation women tend to consume fewer calories and engage in more physical activity; they are judged to be more attractive, express greater preferences for masculine and symmetrical men, and experience increases in sexual desire for men other than their primary partners. Some of these cycle phase shifts are moderated by partner attractiveness and interpreted as strategic responses to women's current reproductive context. The present study investigated changes in sleep across the ovulatory cycle, based on the hypothesis that changes in sleep may reflect ancestral strategic shifts of time and energy toward reproductive activities. Participants completed a 32-day daily diary in which they recorded their sleep time and quality for each day, yielding over 1,000 observations of sleep time and quality. Results indicated that, when the probability of conception was high, women partnered with less attractive men slept more, while women with more attractive partners slept less.

## Introduction

Human females, like nearly all female mammals, experience a very brief window of fertility across the ovulatory cycle, and it is during this time that mating relevant features, perceptions, and behaviors are most likely to affect the genetic quality of a woman's offspring. Because of the reproductive significance of this brief window, it has been hypothesized that women, like other female mammals, will exhibit shifts in their mate preferences, mating behaviors, and attractiveness. The ovulatory shift hypothesis [1], [2] proposes that women will experience systematic shifts in desires and mating-related behaviors when most likely to conceive in order to increase the likelihood of reproducing with genetically fit partners and decrease the likelihood of reproducing with genetically less fit partners.

Dozens of studies have produced evidence that women prefer more masculine and more symmetrical men as sexual partners at high fertility relative to low (see [3], for a review of ovulatory effects on women's mate preferences). Other studies have suggested that women's physiological features may change perceptibly as a function of fertility across the cycle, with evidence that men prefer the scents [4], [5], the voices [6], [7], and the faces and bodies [8] of women in the ovulatory phase of their cycles compared to women in the non-ovulatory phases (see [9] for a review). Some of the studies examining men's preferences for fertile women suggest the possibility that it is women's behaviors, and not only physiological features, that shift with fertility. For example, Miller, Tybur, and Jordan [10] documented that professional lap dancers earned significantly higher tips during the ovulatory phase of their cycles than at other phases, which may be the result of changes in women's appearance, voice, or scent, or may be the result of changes in women's flirtatious or seductive behavior. Additionally, the voice preferences documented by Bryant and Haselton [7] were confined to social speech acts, as opposed to non-verbal sounds, suggesting that men were not responding to a simple physiological change that affects the voice.

Changes in women's behaviors as a function of ovulatory phase have also been documented in other domains, and have been interpreted as either increasing women's likelihood of obtaining a desirable sexual partner or decreasing their likelihood of encountering an undesirable sexual partner. For example, women have been judged to take greater care in their appearance [11], [12], to show more interest in attending social gatherings where they are likely to meet desirable men [13], and to selectively flirt with men who possess proposed cues to genetic quality [14] at high fertility relative to low. Relatedly, women have been shown to decrease rape-related risky behaviors [15] and increase hand-grip strength in response to hypothetical rape scenarios [16] at high fertility compared to low (but see [17], for a discussion of the effectiveness of these behaviors). Women have also been shown to express greater sexual disgust sensitivity [18] and to selectively reduce affiliation with close male kin, but not close female kin [19] at high fertility relative to low. These patterns have been interpreted as reducing the possibility of reproductively costly scenarios such as inbreeding. Finally, women have been shown to exhibit behavioral shifts related to fertility that appear to be more distally related to reproductive strategies, such as consuming fewer calories at high compared to low fertility [20]. This pattern has been provisionally interpreted as a reordering of energetic demands to divert time and energy resources away from somatic maintenance and toward reproductive effort.

In the medical literature, researchers have investigated the possibility that there are changes in sleep patterns across the ovulatory cycle, but have failed to find any significant patterns of cycle phase on sleep time [21]–[23]. We show that if one adopts an evolutionary framework of potential reproductive costs and benefits, it is possible to uncover hitherto undocumented but meaningful effects of fertility on sleep patterns.

### Features of Women's Romantic Partners as an Important Covariate

An important feature of many of the documented ovulatory changes in women's desires, behaviors, and appearance is that they are context-dependent and, in particular, that the features of a woman's romantic partner has a significant effect on how ovulation will influence her. The dual mating hypothesis [24] suggests that the evolutionary context of long-term pairbonding in humans would have resulted in ancestral women having to face substantial tradeoffs in romantic and sexual partner choice. While the best outcome may have been to form a long-term pairbond with a man high in both genetic quality and cooperative partner qualities, not all men would be equally likely to possess all of these traits, nor would the few men who possessed the combination of such traits be able to simultaneously form long-term exclusive pairbonds with all the women who desired them. The dual mating hypothesis therefore proposes that among those women whose partners were relatively high in investing qualities, but relatively low in genetic quality, those who maintained their primary pairbond while opportunistically engaging in extra-pair sex with men of high genetic quality at high fertility would have had greater reproductive success on average than those who did not pursue this strategy.

This theoretical framework has proven useful in predicting nuanced patterns in women's desires and behaviors across the ovulatory cycle. For example, women who rate their partners as less sexually attractive (a hypothesized indicator of genetic quality) have been shown to experience an increase in sexual desire for men other than their current partners when fertile, whereas this shift is absent or minimized among women who rate their partners as high on sexual attractiveness (e.g., [2], [13], [14], [25]). Similarly, women who rate their partners as less sexually attractive feel less close to and more critical of their romantic partners when fertile than at other times in the menstrual cycle [26]. Partner sexual attractiveness also interacts with fertility to predict women's reports of their partners' mate guarding (e.g., jealousy and vigilance) and positive mate retention (e.g., expressions of love and commitment) behaviors [13], [24]. Fessler [20], in his review of periovulatory decreases in calorie consumption and increases in ranging activities (distances traveled in a day), suggested that such shifts may be specifically related to increased preparedness for opportunistic extra-pair mating, as opposed to mating opportunities with one's primary partner, arguing that increased ranging and reduced time spent foraging are unnecessary for maximizing mating opportunities with a primary partner with whom a woman presumably spends a lot of time.

### Sleep Patterns and Fertility

Sleep, like eating and foraging, is likely to serve a somatic maintenance function, although the precise role it plays continues to be debated (see [27] and [28] for a review of sleep's role in memory consolidation, [29] for a discussion of the role of sleep in avoiding predation, [30] for an examination of the necessity of sleep to replenish energy, and [31] for a discussion of the role of sleep in energy conservation). As such, we might expect sleep to be subject to the same sort of reordering at high fertility as are eating and ranging activities. Based on household patterns and sleeping arrangements reported in the ethnographic literature, it is reasonable to assume that individuals throughout most of our ancestral past shared a sleeping space with their primary sexual and social partner, and that much of one's sexual activity with their primary partner would have taken place during sleep times when both partners were likely to be together (see, e.g., [32]–[38]; but also see [39] for a different pattern). We might, therefore, expect any ovulatory effects on sleep to be more relevant to advancing or avoiding conception with one's primary partner than actively seeking extra-dyadic mating opportunities.

### Predictions

We predicted that women would decrease their sleep time at high fertility, when the potential reproductive benefits to ancestral women of reallocating that time toward mating-related activities with her primary partner (i.e., sexual intercourse) would have been highest. We further predicted that this effect would be moderated by a woman's ratings of her primary partner's sexual attractiveness, as the reproductive benefit that would have been gained in our ancestral history by engaging in mating opportunities with a partner who displayed indicators of good genes would have been greater than that which could have been gained by engaging in mating opportunities with a partner with lower sexual attractiveness.

## Materials and Methods

### Participants

This study was approved by the institutional review board at California State University, Fullerton. Participants signed an IRB approved written consent form to participate in the study. The original data presented here is available via the public repository eScholarship, http://escholarship.org/uc/item/4jz9p4fg. Participants were 63 normally-cycling women who had not taken hormonal contraceptives within the previous three months. All women were students at California State University, Fullerton (mean age = 22.41, *SD* = 3.45) and received extra credit for participation. Data were collected in two waves during a single academic year. There were no significant differences between the groups on any variables measured, thus both groups were combined for tests of the predictions. Of these 63 participants, 8 were omitted for atypical or irregular menstrual cycle lengths (i.e., an average cycle length outside the range of 24–35 days or a self-reported irregular menstrual cycle), and 16 were omitted for reporting that they suffered from frequent or chronic sleep problems. Of the remaining 39 participants, 19 identified as currently involved in a romantic relationship. The majority of participants self-identified as either White (30%) or Hispanic (30%), with 17.5% identifying as interracial, 7.5% as African American, 5% as Asian, and 5% as “other.”

### Procedure

Participants attended a 30 minute introductory session in which they were given a 32-day sleep diary and written and verbal instructions on how to fill out the diary. Diary entries were collected every 7 days to ensure that participants did not attempt to complete the diary from memory, and participants were encouraged to leave blank any days they missed. Participants reported the time they fell asleep each night and the time they woke up each day, as well as the total number of waking minutes during the night and total number of sleeping minutes during the day (e.g., naps). They also rated the quality of their sleep each night on a scale of 1 (“below average”) to 5 (“above average”). Participants were also asked to record any factors that may have substantially affected their ability to sleep – either positively or negatively – and whether or not they felt sick on that day. Women also indicated any days on which they were menstruating during the study.

After completion of the 32-day sleep diary, participants attended a final session in which they filled out a return survey. The return survey included a number of items related to participants' sleep patterns, menstrual cycles, relationship status, and partner attractiveness.

### Variables

After collecting the diaries and surveys, total sleep time and fertility phase were calculated for each day. Total sleep time was the amount of time between falling asleep at night and waking in the morning, minus any waking during the night, plus any napping during the day (*M* = 7.69 hours, *SD* = 1.9 hours, *N* = 1,074 observations).

To calculate conception probability, we determined the cycle day of each observation for each woman using a standard 29-day cycle length [40] and dates of menstrual onset. If a woman reported two periods within the diary timeframe, we used this confirmed cycle length to determine cycle day. Once a cycle day was determined for each observation, we used the estimates reported in Wilcox, Dunson, Weinberg, Trussell, and Baird [41] to assign a conception probability (Range = 0 to 0.094) to each observation (see Appendix S1 for complete table).

Participants' descriptions of factors affecting their ability to sleep were rated by a coder blind to the purpose of the study. Each comment was rated on a scale from −2 (indicating features disruptive to sleep, e.g., loud noises, stress, illness) to +2 (indicating features conducive to sleep, e.g., quiet room, comfortable bed, relaxed feeling). Women did not comment on all diary days (on average, women commented on only 22% of the days for which they filled out the diary), and were slightly more likely to report negative sleep factors (*M* = −0.49, *SD* = .98, *N* = 279). In order to retain as many observations in our analyses as possible, days for which there was no comment were coded as the average value for all coded days. This variable was labeled “sleep environment” (*M* = −0.49, *SD* = .51, *N* = 1,031). Days for which participants included comments related to something other than the sleep environment were coded as missing (*N* = 43 observations).

Women in relationships rated the physical and sexual attractiveness of their current partner using two items in the survey, “How *physically* attractive would others rate your partner?” and “How *sexually* attractive would others rate your partner?” Each variable was rated from 1 (“not physically [sexually] attractive”) to 9 (“very physically [sexually] attractive”). These variables were summed to create a single measure of partner attractiveness (α = .924), with a possible score of 2–18 (*M* = 14.05, *SD* = 2.17, *Range* = 10 to 18).

## Results

### Analysis

Analyses were conducted in Stata 13. Because our data include crossed factors (i.e., factors that vary by subject and factors that vary by observation), we used multi-level regression techniques [42]. The models were maximum likelihood regression models with random effects, using individual participants as clusters (*n* = 39 clusters in the models including all women, and *n* = 19 clusters in the models including only women in relationships). This model allows for the maximum degrees of freedom as it uses the number of observations, rather than the number of clusters, as the denominator [43]. The models included both Level 1 (within participant) and Level 2 (between participant) covariates. In the analyses including all women (*n* = 39), there were a total of 1,030 observations of sleep time (*M* = 26.4 per woman; *Range* = 11 to 32) and 1,008 observations of sleep quality (*M* = 25.8 per woman, *Range* = 11 to 32). In the analyses including only those women in relationships (*n* = 19), there were a total of 500 observations of sleep time (*M* = 26.3 per woman; *Range* = 11 to 31) and 482 observations of sleep quality (*M* = 25.4 per woman; *Range* = 11 to 31).

### Sleep Environment and Conception Probability

**Figure 1:**
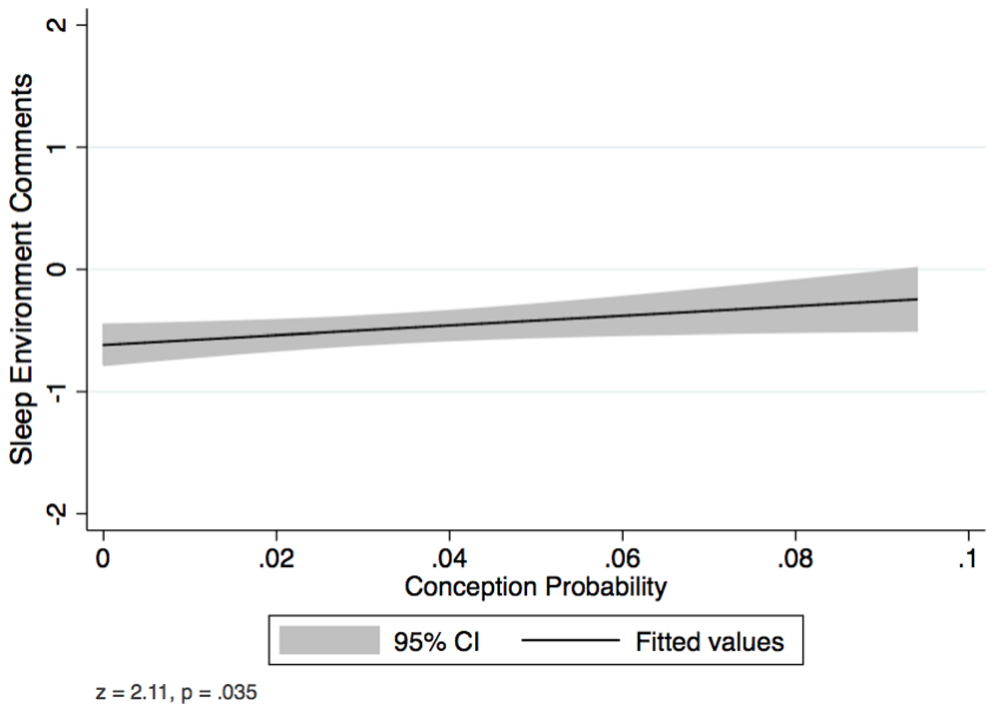
Effect of conception probability on women's reports of their sleep environment. Comments were coded from −2 (factors disruptive to sleep) to 2 (factors conducive to sleep). Multi-level regression performed only on observations that included a valid comment regarding sleep environment (*N* = 279 observations, *n* = 35 women).

The question regarding “sleep environment” was included in the questionnaire to control for extrinsic variation in the sleeping environment that could independently affect sleep time and/or sleep quality. Upon investigation of the variable, we found an unpredicted relationship with conception probability, such that as conception probability increased, women were significantly less likely to report negative sleep factors (*z* = 2.11, *SE* = 1.77, *p*  = .035, *N* = 279 observations, *n* = 35 clusters, see Figure 1). This effect was the same whether we used only those observations that included a valid entry for this question (N = 279) or whether we included observations that had been coded as the mean response (N = 1,031). Further analyses revealed no evidence that this variable had a mediating effect between our predictor and outcome variables, but the inclusion of sleep environment as a control variable did reliably increase the predictive power of all of the models, without changing any patterns of effects. Sleep environment was therefore retained in all analyses to control for variation in sleep time and quality due to extrinsic qualities of the sleep environment.

### Conception Probability and Sleep Time and Quality

In models looking only at the effects of conception probability and sleep environment (*N* = 1,008–1,030 observations), there was no main effect of fertility on either sleep time (*z* = 0.97, *SE* = 143.63 *p*  = .333) or sleep quality (*z* = −0.20, *SE* = 1.11 *p* = .841). Sleep environment significantly and positively predicted both sleep time (*z* = 3.03, *SE* = 10.35, *p* = .002) and sleep quality (*z* = 8.90, *SE* = 0.08, *p*<.0005).

### Conception Probability, Partner Attractiveness, and Sleep Time

**Figure 2:**
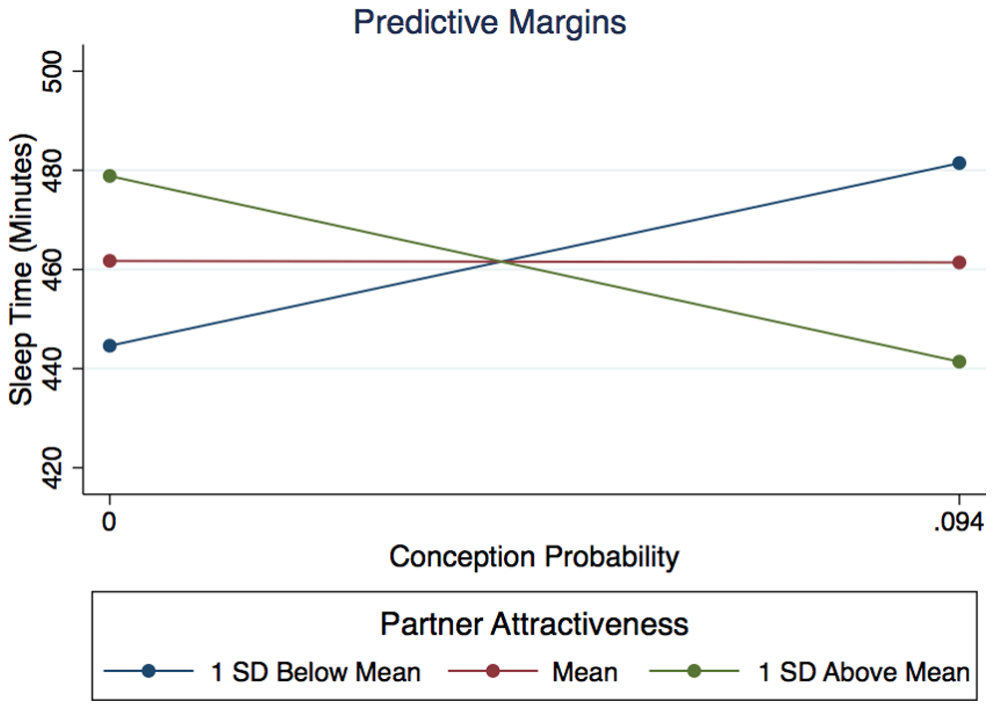
Interaction of conception probability and partner attractiveness on sleep time. Each line represents the marginal effect of conception probability on sleep time at a different level of partner attractiveness.

We conducted a second set of analyses to examine the effects of conception probability, partner attractiveness, and the interaction of those two variables on sleep time and quality. Sleep environment was included as a control variable and the samples included 19 women in relationships (*N* = 482–500 observations). Both conception probability (*z* = 2.93, *SE* = 891.72, *p* = .003) and sleep environment (*z* = 5.50, *SE* = 8.16, *p*<.0005) positively predicted sleep time when controlling for the independent effects of partner attractiveness and the interaction of partner attractiveness and conception probability. Partner attractiveness had no main effect on sleep time (*z* = 1.55, *SE* = 5.21, *p* = .120). There was a significant interaction of conception probability and partner attractiveness on sleep time (*z* = −2.98, *SE* = 62.57, *p* = .003), indicating that conception probability affects sleep time differently depending on the attractiveness of one's partner (see Figure 2).

To further examine the relationship between conception probability, partner attractiveness, and sleep time, we tested the marginal effects of conception probability on sleep time at different levels of partner attractiveness. Among those women who rated their partners as most attractive (i.e., 1 *SD* above the mean), there was a significant negative effect of conception probability on sleep time (see Table 1). In other words, women with more attractive partners were sleeping less when the probability of conception was higher. Among those women who rated their partners as least attractive (i.e., 1 *SD* below the mean), there was a significant positive effect of conception probability on sleep time. In other words, women with less attractive partners were sleeping more when the probability of conception was higher. Among those women who rated their partners as intermediate in attractiveness there was no significant relationship between conception probability and sleep time.

**Table 1:** Marginal effects of conception probability on sleep time at each level of partner attractiveness.

Partner Attractiveness Rating	Marginal Effect of Conception Probability on Sleep Time *z* (*SE*)
**1 ** ***SD*** ** Below Mean**	**2.02 (194.23)** *
Mean (14.05)	0.00 (139.59)
**1 ** ***SD*** ** Above Mean**	**−2.09 (190.82)** *

*p<.05.

We also examined the marginal effects of partner attractiveness at different levels of conception probability. Partner attractiveness had no marginal effects on sleep time at any level of conception probability (all *ps*>.160).

### Conception Probability, Partner Attractiveness, and Sleep Quality

**Figure 3:**
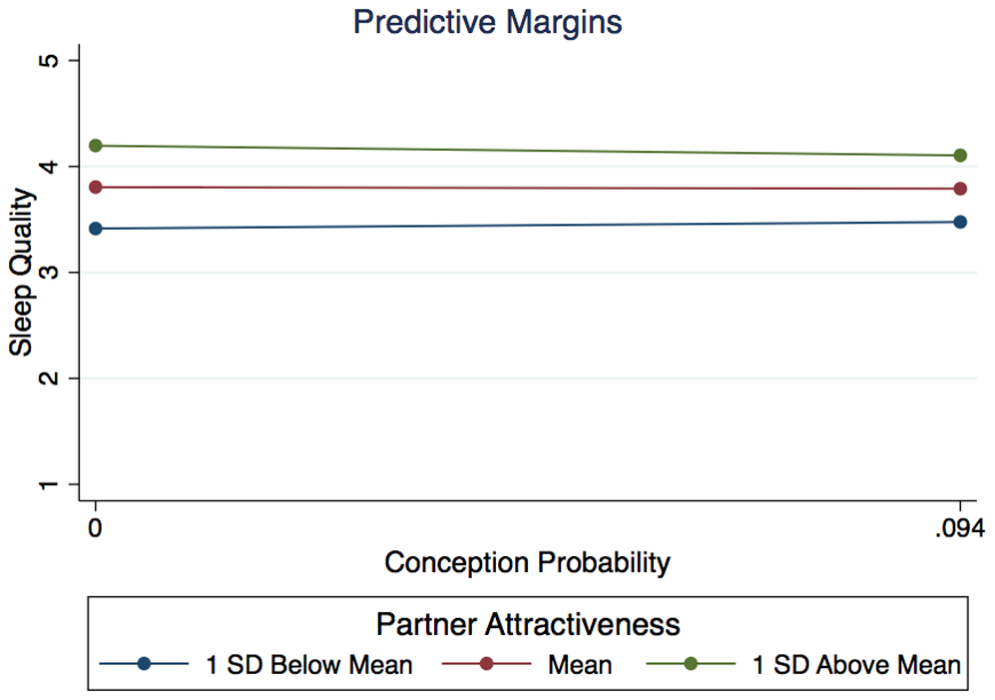
Effects of conception probability and partner attractiveness on self-reported sleep quality. Each line represents the marginal effect of conception probability (all non-significant) on sleep time at a different level of partner attractiveness. The different heights of each line illustrate the significant main effect of partner attractiveness on sleep quality.

We next looked at the effects of fertility and partner attractiveness on self-rated sleep quality. Fertility alone was not significantly related to sleep quality (*z* = 0.71, *SE* = 7.50, *p* = .480), and there was no interaction between partner attractiveness and conception probability on sleep quality (*z* = −0.74, *SE* = 0.53 *p* = .461). There was a main effect of partner attractiveness (*z* = 3.25, *SE* = 0.06, *p* = .001) such that those women who rated their partners as more attractive also rated the quality of their sleep better (see Figure 3). Sleep environment also significantly and positively predicted self-reported sleep quality (*z* = 10.31, *SE* = 0.07, *p*<.0005).

## Discussion

We predicted that women would sleep less when conception probability was high, and that this effect would be moderated by their partner's attractiveness. Results were partially consistent with this prediction and lend support to the general hypothesis that women's mating psychology may reflect adaptations designed to strategically adjust the time and energy allocated toward somatic maintenance during high fertility [20].

### Sleep Time

When looking only at the effect of fertility on sleep time, we, like previous researchers, failed to find a significant relationship. However, when we included the sexual attractiveness of the women's primary partners as a covariate in the model, we found a significant positive effect of conception probability on sleep time, such that in general, as conception probability increased, so did the amount of time sleeping. In accordance with our predictions, there was also a significant interaction with partner attractiveness, suggesting that the effect of conception probability on women's sleep time is moderated by the attractiveness of her current partner. Women who rated their partners as relatively less attractive (1 *SD* below the mean) experienced a significant *increase* in sleep time at higher fertility, whereas women who rated their partners as relatively more attractive (1 *SD* above the mean) experienced a significant *decrease* in sleep time at higher fertility. Women who rated their partners as intermediate in attractiveness did not experience a fertility-related shift in sleep time.

One interpretation of this pattern is that it suggests divergent reproductive strategies based on the qualities of a woman's current partner. For example, it is possible that in our ancestral past women with more attractive partners strategically reduced their sleep time when the probability of conception was highest, that is, when the energy that would otherwise have be conserved could have net potentially greater returns for ancestral women by being invested in mating activities, such as sexual intercourse.

Similarly, it is plausible that the fertility-related increase in sleep time we documented among women with less attractive partners reflects a conception-avoidance strategy if, among ancestral women, extending one's sleeping time might have reduced the possibility of sexual interaction with one's partner during the night. Such an interpretation, although tentative, has some precedence. Kruger and Hughes [44] documented that among cohabiting couples, when no sex has taken place, women are more likely to fall asleep first. They interpreted this pattern as a conception-avoidance strategy, but did not examine either the effects of fertility or partner attractiveness.

Another potential source of the pattern of effects documented in this study is that increased sleep among women with less attractive partners reflects greater energy expenditures during the day. It has been demonstrated by many previous studies that women with less attractive partners are those who may be most likely to engage in extra-pair mate seeking (e.g., [2], [13], [14]). Fessler [20] further argued that extra-pair mate seeking may be the underlying motivation for documented increases in ranging behavior and other physical activities among women at high fertility. It is reasonable to think that these behaviors are most likely to be those that occur during waking hours, and not when a woman is sleeping next to her primary partner, thus allowing for a possible increase in sleep time at night.

Unfortunately, in the current data set, we are unable to further test these possible explanations for the patterns documented. Future research should investigate these possibilities by examining additional variables. Most direct would be to assess sexual desire or behavior. If ancestral women with less attractive partners were sleeping more at high fertility in order to avoid conception, we might expect women with less attractive partners to also experience less sexual desire for their partners and have sex less often at high fertility than those women who rate their partners as more sexually attractive. Although women in the current study were asked about sexual behavior, there were too few responses to adequately test this prediction with these data. In addition, assessing daily activity levels could be informative. If women with less attractive partners benefitted by engaging in extra-pair mate seeking behaviors that used more energy during the day, we should see that increased sleep time is associated with behaviors such as increased ranging or social involvement.

### Sleep Quality

As with sleep time, when examined alone, conception probability had no significant relationship to sleep quality, and there remained no evidence of a main effect of fertility on sleep quality when including partner sexual attractiveness as a covariate in the model. We did, however uncover a significant main effect of partner attractiveness on the quality of women's sleep. That there is a general relationship between partner attractiveness and sleep quality is unsurprising, and can likely be attributed to any number of covarying factors. For example, the woman's own health, stress levels, social status, or access to resources all may affect both her ability to obtain a physically attractive partner and her overall ability to enjoy a good night's sleep.

### Sleep Environment

Another unpredicted finding to come out of this study was that women's reports of the sleep environment itself changed over the ovulatory cycle, such that women reported significantly fewer negative sleeping conditions when conception probability was higher. Although conception probability significantly predicted women's reports of their sleep environment, and their sleep environment, unsurprisingly, predicted both sleep time and quality, there was no evidence of statistical mediation, that is, conception probability does not appear to be affecting sleep time or quality *through* sleep environment. Although we cannot further examine this effect with our current data, there are a number of intriguing possibilities that could explain this pattern. First, changes in women's *behavior* might result in different sleep environments at different points in the cycle. For example, it may be that women are more conscientious about closing windows, adjusting the heat, or otherwise ensuring a more peaceful sleep environment at high fertility than at low fertility. Secondly, intrinsic changes in women's ability to sleep may affect their *perception* of their sleep environment. For example, it may be that women are less aware of ambient noise, temperature fluctuations, or other potential distractions during high fertility than they are at low fertility. A third possibility is that it is women's *sensitivity* to their environment, but not the environment itself or their ability to perceive the features in the environment, which fluctuate. For example, women may be just as aware of ambient noise at high fertility as they are at low fertility, but find it less distracting and thus fail to report it in their diaries. Further research may be able to discern whether this pattern is a simple byproduct of physiological changes at high fertility or represents a strategic shift in behavior or perception.

### Limitations

This study suffers from some additional unavoidable limitations. Since our dependent measures are all based on self-report, it is possible that the data include errors, misrepresentations, or variations in interpretation, however there is no reason to believe that any such error would vary systematically with either conception probability or partner attractiveness. It is also probable that unassessed variables, such as work and school demands, could have a significant effect on women's sleep patterns, but these, too, are not expected to vary systematically with conception probability or partner attractiveness.

### Conclusion

While the current study cannot be construed as conclusive evidence for any specific ancestral adaptation, we believe that the pattern of results documented is important and relevant to our growing understanding of women's sexuality and behavior. The data reported here provide evidence of systematic shifts in women's sleep patterns as a combined function of fertility and the qualities of a woman's primary partner. In general, we found that women with more sexually attractive partners slept less when the probability of conception was higher, while women with less attractive partners slept more when the probability of conception was higher. We have tentatively interpreted these findings as reflections of divergent reproductive strategies among women based on the qualities of their primary partners. For women with more attractive partners, we suggest that the decreased sleep at high fertility may reflect a strategic shift from somatic maintenance to reproductive effort when conception is most likely. In terms of the results for women with less attractive partners we have proposed several possible explanations that warrant further research, including the possibility that women with less attractive partners are strategically avoiding conception. These results will hopefully lead to future studies to further investigate the subtle reproductive strategies of women and their effects on everyday health and wellbeing.

## Supporting Information

Appendix S1 **Daily Conception Probability.** Daily conception probability for women with regular cycles taken from [41].
(DOC)
