# Supplementary material for: Changes in Sleep Time and Sleep Quality across the Ovulatory Cycle as a Function of Fertility and Partner Attractiveness
Source: PLoS One. 2014 Apr 7;9(4):e92796. doi: 10.1371/journal.pone.0092796 (PMC3977843; doi:10.1371/journal.pone.0092796)
Supplement: Appendix S1 — Daily Conception Probability. Daily conception probability for women with regular cycles taken from [41]. (DOC) [file pone.0092796.s001.doc]

Appendix S1

Daily conception probability for women with regular cycles taken from [41].

| Cycle Day | Conception Probability for Women with Regular Cycles |
| --- | --- |
| 1 | 0.000 |
| 2 | 0.000 |
| 3 | 0.001 |
| 4 | 0.002 |
| 5 | 0.004 |
| 6 | 0.009 |
| 7 | 0.018 |
| 8 | 0.032 |
| 9 | 0.050 |
| 10 | 0.069 |
| 11 | 0.085 |
| 12 | 0.094 |
| 13 | 0.093 |
| 14 | 0.085 |
| 15 | 0.073 |
| 16 | 0.059 |
| 17 | 0.047 |
| 18 | 0.036 |
| 19 | 0.028 |
| 20 | 0.021 |
| 21 | 0.016 |
| 22 | 0.013 |
| 23 | 0.010 |
| 24 | 0.008 |
| 25 | 0.007 |
| 26 | 0.006 |
| 27 | 0.005 |
| 28 | 0.005 |
| 29 | 0.005 |
| 30 | 0.006 |
| 31 | 0.006 |
| 32 | 0.007 |
| 33 | 0.007 |
| 34 | 0.008 |
| 35 | 0.009 |
| 36 | 0.010 |
| 37 | 0.010 |
| 38 | 0.011 |
| 39 | 0.012 |
| 40 | 0.013 |
